# High-Purity Quantum Emission from an Au_24_(S-CH_2_Ph‑^*t*^Bu)_20_ Nanocluster at Room Temperature

**DOI:** 10.1021/acsnano.6c05162

**Published:** 2026-05-14

**Authors:** Abhrojyoti Mazumder, Ece Gunay, Guiying He, Zhongyu Liu, Sebastian Calderon, Lianshun Luo, Yitong Wang, Mircea Cotlet, Elizabeth C. Dickey, Rongchao Jin, Linda A. Peteanu

**Affiliations:** † Department of Chemistry, 6612 Carnegie Mellon University, Pittsburgh, Pennsylvania 15213 United States; ‡ Department of Materials Science and Engineering, Carnegie Mellon University, Pittsburgh, Pennsylvania 15213 United States; § Center for Functional Nanomaterials, 8099 Brookhaven National Laboratory, Upton, New York 11973, United States

**Keywords:** atomically precise gold nanoclusters, photoluminescence, photon antibunching, single-photon emission, room temperature, quantum emitters

## Abstract

Atomically precise gold nanoclusters have garnered significant attention for their diverse applications, ranging from biological labeling to optoelectronics. Their potential in optical quantum computing, which calls for ideal single-photon sources, has recently become a key area of interest. In the current work, we use photon antibunching experiments to explore the single-photon emission efficiency of atomically precise Au_24_ nanoclusters protected by 4-*tert*-butylbenzyl mercaptan ligands (Au_24_(TBBM)_20_). This cluster exhibits quantum emission with good photostability and without any observable blinking or spectral drift at room temperature under an inert gas atmosphere, with antibunching dips (g^2^(0)) as low as 0.07 in the solid state or, equivalently, a single-photon purity of 93% under time-gated conditions. Transient absorption and time-gated antibunching studies reveal that the short emission lifetime of this cluster and its high photoluminescence quantum yield in the solid state play critical roles in enhancing the emitted single-photon purity. This research advances the understanding of single-emitter behavior in atomically precise gold nanoclusters, contributing to the development of stable quantum emitters that are essential for quantum computing and cryptography.

## Graphical abstract

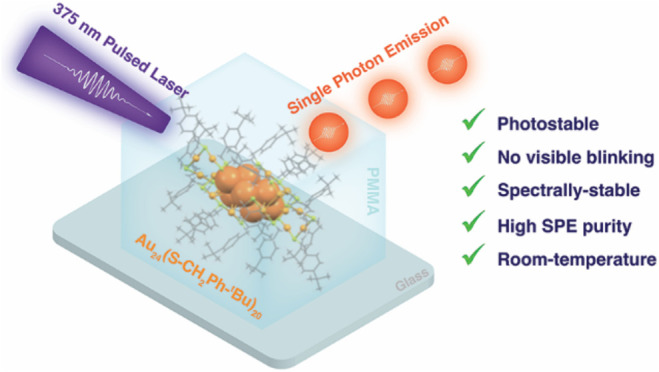

Atomically precise metal nanoclusters (NCs) are a key area of focus in nanoscience research because of their unique quantum-size regime and well-defined structures and have applications in optical, bioimaging, and catalytic processes.^1–6^ NCs consist of a precise number of atoms, usually ranging from dozens to hundreds of atoms, arranged in specific geometric patterns. The photoluminescence (PL) properties of NCs are currently an area of extensive research.^7–14^ In the quantum realm, a new frontier is emerging for nanoscale objects in the field of quantum communication and computing.^15,16^ These applications require materials that are highly photo- and thermally stable and that can emit photons triggered by electrical or light impulses on demand. There are numerous reports of single-photon emission (SPE) from other materials such as nitrogen-vacancy centers in diamonds, carbon nanotubes, and perovskites.^17–25^ However, these are often not defect-free and are not uniform in size, structure, electronic properties, and chemical composition as would be an ideal emitter. In fact, several materials, including diamonds, rely on defects to produce emission.^26,27^ Semiconductor quantum dots (QDs) have also been extensively studied for this application.^28–31^ However, several of these exhibit limiting factors such as blinking (stochastic intermittent emission), long-lived dark states, and nonuniformity of chemical composition and size.^32–34^ In contrast, atomically precise NCs possess several advantages. Importantly, their atomic precision and identical structures remove the uncertainties caused by polydispersity and structural nonuniformity. This allows for a clear determination of structure–property relationships, which is crucial for rational design.^35^ Such NCs also exhibit emission tunable from visible to near-infrared (NIR),^7,36^ including the telecom wavelengths,^37^ depending on composition and structure. Moreover, many NCs exhibit a relatively narrow emission bandwidth and good photoluminescence quantum yield (PLQY), both of which are useful for bright SPE. The PLQY can be enhanced in a solid-state matrix and by rigidifying the surface staple motifs.^38,39^ In summary, the molecular purity of NCs and their absence of defects result in consistent and reproducible emission characteristics. This uniformity is of paramount importance for applications that necessitate precise control over the emitted light, such as single-molecule imaging and SPE.

Despite the growing interest in metal NCs, there has been a significant lack of published studies describing their single-particle optical behavior. In 2002, a pioneering study by Dickson and coworkers reported dendrimer-encapsulated small Ag NCs (Ag_2–8_) at the single-molecule level that exhibited good stability and fluorescence at room temperature.^40^ Over the years, however, only a limited number of studies in metal atom NCs have carefully investigated their photon antibunching,^41^ a key measurement of SPE, with most focusing on DNA-stabilized Ag and Au NCs.^42–45^ Notably, in another study, Dickson and coworkers reported single-stranded (ss) DNA-encapsulated Ag NCs, consisting of 12 cytosine bases (C_12_–Ag_n_).^42^ These NCs demonstrate exceptionally high emission rates without significant intensity fluctuations (blinking) over time scales of 0.1 to 1000 ms, along with outstanding photostability and antibunching. Later, a study by Vosch and coworkers investigated the photophysical properties of NIR-emitting Ag NCs stabilized in ssDNA with 24 cytosines (C_24_–Ag NCs) using single-molecule spectroscopy in the solid state at room temperature.^43^ In this study, the emission maxima, fluorescence decay times, fluorescence intensity, blinking, and photon antibunching were determined simultaneously. Some of the C_24_–Ag NCs exhibited changes in their spectral properties during measurements, such as emission maxima and decay time, which were attributed to variations in the local environment or in the DNA conformations. Although good antibunching was evident, noticeable blinking and spectral drift were also observed at the single-molecule level. Another report by the same lab demonstrated single-molecule detection of DNA-Ag NCs emitting at the NIR I/II border, both in solution and in the solid state at room temperature.^44^ Whereas clear antibunching dips were observed, these NCs exhibit fluorescence intermittency, they are not atomically precise, and they did not exhibit stable emission spectra or long survival times. Sen and coworkers have recently performed solid-state single-particle analysis of DNA-templated Au NCs at room temperature, in which photon antibunching reported; however, these NCs still lack atomic precision and exhibit detectable blinking.^45^

In a recent study, the Patra lab demonstrated photon antibunching in dihydrolipoic acid-protected Ag_29_ NCs, highlighting the potential of chemically well-defined NCs for SPE at room temperature in the solid state.^46^ Furthermore, their research revealed that substituting silver with gold in the Ag_29_ could suppress blinking, enhance photostability, and significantly improve single NC emission by increasing the overall PLQY. However, the photon antibunching dip at zero delay (g^2^(0)) was not quantified, which is important to understanding the emitted single-photon purity. In another study, Wenger and coworkers investigated the photodynamics of glutathione-protected Au_18_(SG)_14_.^47^ Although photon antibunching experiments were not reported, the authors identified evidence of the quantum nature of Au_18_(SG)_14_ at room temperature using fluorescence correlation spectroscopy (FCS) in solution. Inspired by the recent advances (for a compilation see Table S1) and the significance of photon antibunching techniques in exploring quantum behavior, our focus here is to quantify the photon antibunching properties of an atomically precise homometallic Au NC at room temperature in solution and in the solid state, the latter being directly relevant for practical applications.

Efficient SPE is typically achieved in materials that have relatively short radiative lifetimes of nanoseconds or shorter.^32,48,49^ Though there are limited examples of metal NCs exhibiting fluorescence.^50–53^ Au_24_(TBBM)_20_, as previously reported by the Jin group,^54^ is one notable case. Whereas some ensemble-level research has been conducted on Au_24_(TBBM)_20_,^55–57^ there have been no single-cluster studies performed to date. The PL spectra obtained from conventional spectrometry techniques report on the collective properties of all the NCs in a given solution-phase or solid-state sample. These ensemble measurements provide no insight into individual cluster emission behavior, such as blinking properties, single-particle PL lifetimes, photostability, photon antibunching, and the influence of the local environment—parameters that are critical for high-quality SPE.

In this work, we investigate these single-cluster characteristics of Au_24_(TBBM)_20_ both in solution and in the solid state using confocal microscopy at room temperature. We find that the individual NCs display exceptional spectral stability and photostability, showing no detectable blinking at room temperature under an inert gas atmosphere and with single-photon purity as high as 93% in the solid state, under time-gated conditions. This rivals some of the best, comparatively larger, quantum materials that have been reported.^23,25,48,58^ Although some nearly blinking-free efficient SPE quantum materials have been reported^59–62^ and efforts are ongoing to achieve atomic precision,^63,64^ many of them still suffer from rapid photobleaching, spectral diffusion, and fluorescence intermittency (blinking), that collectively hinder their performance in long-time scale measurements at room temperature. In contrast, atomically precise NCs, representing a distinct class of materials, offer a promising pathway toward ideal, intrinsically stable SPE sources.

## Results and Discussion

The atomic structure of Au_24_(TBBM)_20_ features a bitetrahedral Au_8_ (inner) core (Figure 1A) in which two tetrahedra are connected in an antiprismatic manner through two triangular faces (face-to-face).^54^ This Au_8_ core is shielded by four Au_4_S_5_ surface motifs (Figure 1B), forming an overall prolate structure (Figure 1C). To demonstrate that our sample consists of single clusters that are not prone to aggregation at the concentrations used for solid-state single-molecule optical microscopy, aberration-corrected scanning transmission electron microscopy (STEM) imaging of the NCs was performed at 80 kV using a high-angle annular dark-field (HAADF) detector to provide atomic-number contrast. For sample preparation, a ∼100 pM solution of Au_24_(TBBM)_20_ NCs in toluene was drop-cast onto an ultra-thin lacey-lacey-carbon-coated Cu TEM grid and left to air-dry for 2 h to ensure complete solvent evaporation before imaging. A representative image (Figure 1D), acquired with a pixel size of 65 pm, displays well-isolated NCs at the concentration used. Analyzing over 200 NCs (see SI Figures S1 and S2), we found a size distribution with a peak value for the long axis of 1.12 ± 0.3 nm (SI Figure S3). Note that the size distribution is presumably due to different orientations of the rod-shaped Au_24_ (Figure 1E se F), as the values are consistent with the expectations derived from crystallographic data.^54^

**Figure 1:**
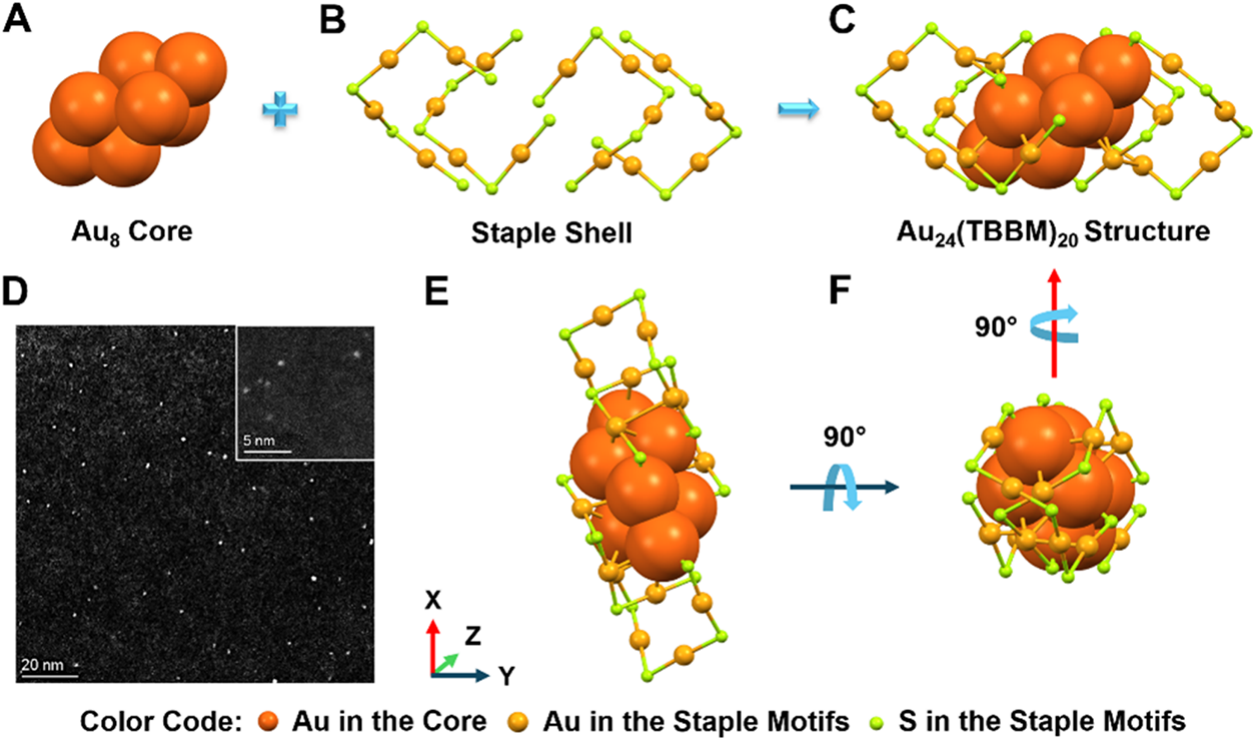
(A–C) Anatomy of the X-ray structure of **Au**_**24**_ **(TBBM)**_**20**_. (D) HAADF-STEM image of the NCs. In the inset: a representative high-magnification HAADF-STEM image. (E, F) Different projections of **Au**_**24**_ **(TBBM)**_**20**_. In all the X-ray images, the carbon tails are omitted for clarity.

### Room Temperature Bulk-Phase Photophysical Studies of Au_24_(TBBM)_20_

#### In Solution

The optical absorption spectrum of Au_24_(TBBM)_20_ in a dilute degassed toluene solution displays peaks at 380, 430, and 500 nm (Figure 2A, black). Its PL spectrum in toluene (Figure 2A, solid-red) exhibits dual emission, with one peak in the visible at 670 nm (PL I) and a second in the NIR (PL II) at 950 nm. The reported PLQY (PL I+PL II) of Au_24_(TBBM)_20_ is ∼3% in toluene.^55^ The PL excitation spectrum for the PL I (Figure 2A, dotted-red) strongly resembles the UV–vis absorption spectrum, particularly in the spectral region ∼350 nm and above. This suggests that these clusters obey Kasha’s rule and that PL I arises from the first excited state of Au_24_(TBBM)_20_.^55^ The lifetime of PL I at room temperature in toluene (Figure 2B,2C) was determined using time-correlated single photon counting (TCSPC) with an instrument response function (IRF) of ∼25 ps. The excitation wavelength (375 nm) was chosen to coincide with that used in the antibunching studies discussed later. First, the initial 15 ns of the decay was fit (see Figure 2B) and parametric bootstrap analysis (with 90% probability) was applied to evaluate the variability of the fitted lifetime parameters (see SI Figure S4A). Two decay components were obtained; τ_1_: 0.8 ns and τ_2_: 4 ns (see Figure 2B). These two decay constants were then used to fit the overall decay, which resulted in a third decay component of τ_3_: 88 ns (see Figure 2C), with τ_avg_: ∼11 ns (amplitude weighted). The results are listed in Table 1. These results are similar to those previously reported in dichloromethane.^55^

**Figure 2:**
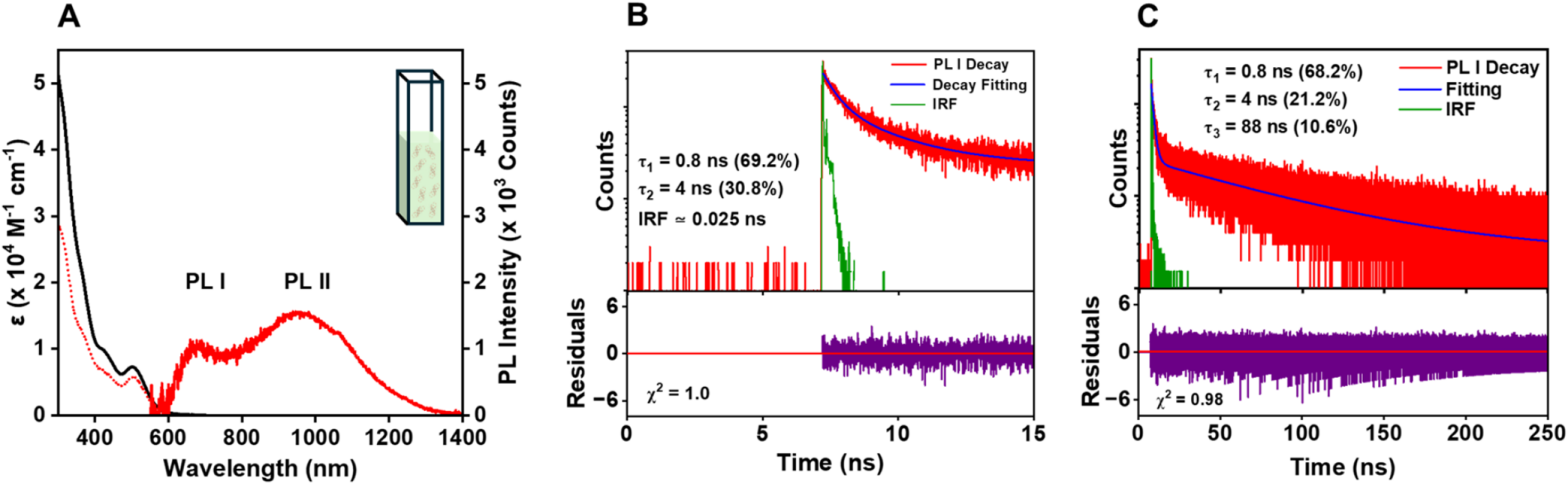
(A) Optical absorption spectra (black line; molar absorption coefficient shown on the left *y*-axis), PL (solid-red lines; intensity on the right *y*-axis), and PL excitation spectra (dotted line; intensity on the right *y*-axis) of **Au**_**24**_ **(TBBM)**_**20**_. The NCs were in degassed toluene. The PL spectra were collected at 0.3 optical density (OD) with 375 nm excitation. (B) Early time zoom-in of bulk-phase TCSPC decay fitting and corresponding fit residuals for **Au**_**24**_ **(TBBM)**_**20**_ in toluene, obtained using a 370 nm excitation laser with a 25 ps pulse width and detection at 650 nm. (C) Overall PL I decay profile of **Au**_**24**_ **(TBBM)**_**20**_ in toluene with fitting residuals.

**Table 1:** Photophysical Data for Au_24_(TBBM)_20_ in Toluene Solution and in PMMA Film

condition	solution	solid
Φ_PL_ (%)	∼2	∼25
PL peaks (eV)	PL I: 1.85	PL I: 1.92
	PL II: 1.30	PL II: weak
PL-I Lifetime (ns)	τ_1_: 0.8 (68.2%)	τ_1_: 1.3 (59.3%)
	τ_2_: 4 (21.2%)	τ_2_: 12 (12.9%)
	τ_3_: 88 (10.6%)	τ_3_: 223 (11.2%)
	τ_avg_: ∼11	τ_4_: 1612 (16.6%)
		τ_avg_: ∼295

#### In Solid Film

For solid-state measurements, polymethylmethacrylate (PMMA) was used as a matrix for the NCs. The absorption (Figure 3A, black) and PL excitation (Figure 3A, dotted-blue) spectra in this matrix are similar to the corresponding spectra in toluene. However, compared to the cluster in toluene, the solid-state sample shows only a single dominant emission peak (PL I, Figure 3A, solid-blue) that is blue-shifted by 25 nm and has a dramatically enhanced PLQY of ∼25%.^55^ To fit the emission decays, measured using TCSPC with an IRF of ∼25 ps, the first 15 ns was fit (see Figure 3B) and then parametric bootstrap analysis (with 90% probability) was applied to evaluate the variability of the resulting parameters (see SI Figure S4B). Two decay components were obtained: τ_1_: 1.3 ns and τ_2_: 12 ns (see Figure 3B). These two decay constants were then used to fit the overall decay. This resulted in two additional components of τ_3_: 223 ns and τ_4_: 1612 ns (see Figure 3C), with τ_avg_: ∼295 ns (amplitude weighted). These results are compiled in Table 1. Lengthening of the excited-state lifetime has been reported in temperature-dependent PL studies of NCs and was attributed to suppression of the fast nonradiative relaxation when molecular motion is restricted.^7,39^ Note that no IRF-limited component is observed in the fluorescence lifetime decay at the excitation wavelength used, meaning that no fast emission quenching processes are evident in these dynamics. Such processes could include biexcitation formation, exciton–exciton annihilation, and nonradiative decay such as that caused by energy transfer to a non-emissive defect state.

**Figure 3:**
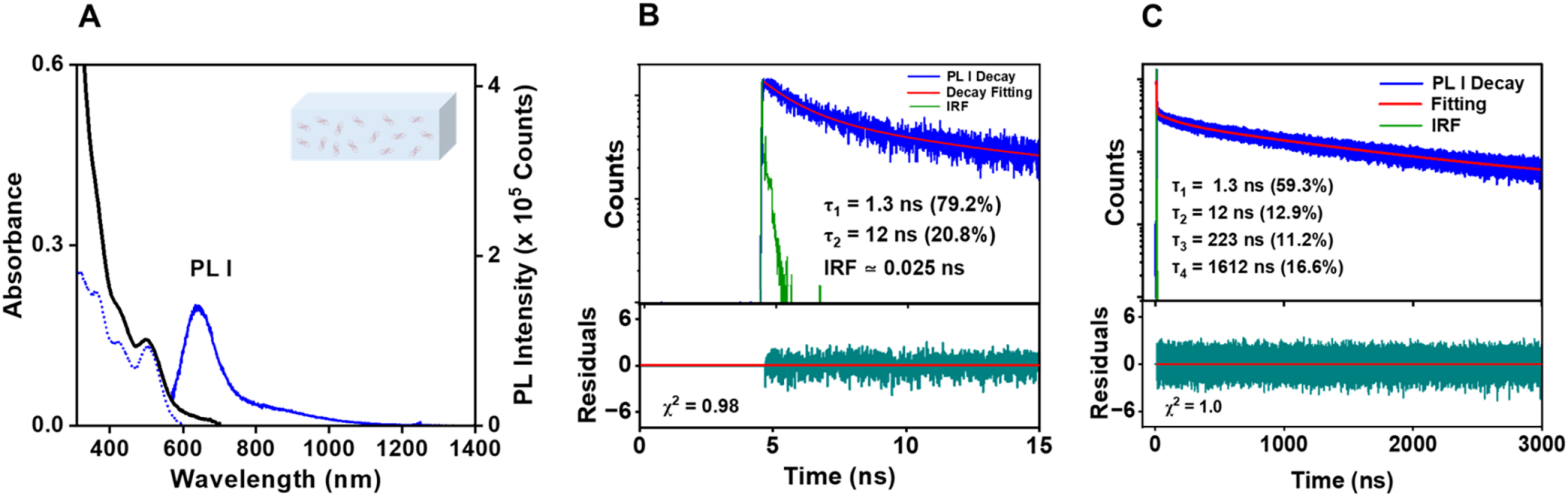
(A) Optical absorption spectra (black line; absorbance shown on the left *y*-axis), PL (solid-blue; intensity on the right *y*-axis), and PL excitation (dotted-blue; intensity on the right *y*-axis) of **Au**_**24**_ **(TBBM)**_**20**_. The NCs were embedded in PMMA under air. PL spectra were collected at 0.3 OD with 375 nm excitation. (B) Early time zoom-in of bulk-phase TCSPC decay fitting and corresponding fit residuals for **Au**_**24**_ **(TBBM)**_**20**_ in PMMA under air, obtained using a 370 nm excitation laser with a 25 ps pulse width and detection at 650 nm. (C) Overall PL I decay profile of **Au**_**24**_ **(TBBM)**_**20**_ embedded in PMMA under air with fitting residuals.

### Room Temperature Single-Photon Emission (SPE) Studies

Photon antibunching is a common technique used in confocal microscopy to confirm that the emission originates from a single source rather than a group of emitters. Photon antibunching was first demonstrated in 1977 through resonance fluorescence experiments on sodium atoms in a low-density atomic beam.^65^ The study provided the first direct observation of photon antibunching in resonance fluorescence, offering clear evidence of the quantum nature of light and atomic quantum jumps beyond the semiclassical explanation. However, its quantitative accuracy was limited because fluorescence sometimes originated from more than one atom, scattered laser light introduced noise, and several corrections were needed, preventing a perfect match with single-atom theoretical predictions. In a study in the early 90s,^66^ single-molecule spectroscopy in p-terphenyl crystals doped with pentacene dye molecules revealed truly quantum-mechanical effects or photon antibunching without being influenced by transit time or motion effects, providing a powerful method to probe molecular dynamics at the single-emitter level. However, the antibunching signal was constrained by background fluorescence and intersystem crossing into triplet states, necessitating careful experimental control of these decay pathways. In 2000, another study demonstrated that on-demand single photons could be extracted from individual terrylene molecules embedded in thin, sublimed crystalline p-terphenyl platelets using photon antibunching measurements at room temperature.^67^

Antibunching measurements are conducted in a Hanbury Brown-Twiss (HBT) setup. For our single-photon emission measurements, we utilized a custom-built confocal instrument with a 375 nm pulsed excitation laser operating at a 2.5 MHz repetition rate (i.e., 400 ns between pulses), capable of detecting emission in the visible range (400–700 nm). We used a 390 nm dichroic/400 long-pass (LP) with a 680 ± 50 nm bandpass (BP) filter to minimize laser and solvent Raman scattering. We used eq 1 to fit all the antibunching data,^25^

1 $$g^{2}\left(\tau \right)=\left[p_{0}-p_{\tau }\right]\exp^{\left(-\left|\tau \right|/\tau_{d}\right)}+p_{\tau }\sum_{i=-n_{peaks}}^{n_{peaks}}\;\exp^{\left(-\left|\tau -\frac{i}{f}\right|/\tau_{d}\right)}$$

in which *g* ^2^(τ) is the second-correlation function at delay τ. *p*_0_ and *p*_τ_ are the amplitude for the central peak and the side peak respectively, “*i*” is the peak number, “*f*” is the repetition rate of the pulsed laser, and τ_d_ is the average lifetime of the emitter.

#### 2.2.1 Solution-Phase SPE

Photon antibunching can be observed in solutions by using the FCS technique. FCS measures the diffusion of fluorophores by monitoring the fluctuations in the photon intensities within the focal volume. A rise in the FCS signal at early times is an indication of antibunching.^68^

To analyze the FCS curve, we used the “pure diffusion model” (eq 2) with additional terms for the antibunching effect, as described below^47,69^

2 $$G\left(\tau \right)=\frac{1}{N}\left(1-\frac{1}{N_{\mathrm{emi}}}\;e^{\left(-\tau /\tau_{A}\right)}\right){\left(1+\frac{\tau }{\tau_{D}}\right)}^{-1}{\left(1+\frac{\tau }{\omega^{2}\tau_{D}}\right)}^{-1/2}$$

where *G*(τ) is the correlation function, *N* is the average number of molecules in the focal volume, τ_D_ is the diffusion time, ω (= *z*_*0*_/*r*_0_) characterizes the dimension of the focal volume with *z*_0_ and *r*_0_ being the axial and radial dimensions of the focal volume, *N*_emi_ is the number of emitting species per molecule, and τ_A_ is the antibunching time.

We also attempted to fit the data by including an additional triplet term $\left(1+\frac{T_{\mathrm{DS}}}{1-T_{\mathrm{DS}}}\;e^{\left(-\tau /\tau_{\mathrm{DS}}\right)}\right)$ to the overall product in eq 2 (where *T*_DS_ is the fraction of emitters in the triplet state, i.e., “triplet fraction” and τ_DS_ is the triplet lifetime) (SI Figure S6). However, we found a very small triplet fraction (∼7–10%) within our working power range (1.6–13.7 μW). For that reason, we utilized the pure diffusion model with an antibunching term (eq 2) to fit our FCS data and to evaluate the effect of laser power on the antibunching time (τ_A_).

Laser-power-dependent FCS measurements were conducted on a ∼100 pM toluene solution of Au_24_(TBBM)_20_, with the results fitted using eq 2. First, the linearity of the total emission signal with laser power was confirmed within our working power range of 1.6 μW to 13.7 μW (Figure 4A), which likely rules out the possibility of biexciton formation in isolated clusters in this excitation power regime. Also, power-dependent PL lifetime decay measurements yielded similar decay constants (see SI Figure S7), indicating the absence of intracluster annihilation processes. In all cases, the early time dip in the correlation curve indicates that the NCs exhibit quantum emission or antibunching (Figure 4B,C). With increasing laser power, the antibunching dip observed on the nanosecond time scales becomes slightly narrower due to a shortening of the time frame over which antibunching takes place (τ_A_) (see SI Figure S8A). No noticeable change is observed in the diffusion time (τ_D_) (SI Figure S8B) as this depends on the molecular size, solution viscosity, and the confocal volume, rather than on the excitation intensity, for powers below the power saturation limit.^47,70^

**Figure 4:**
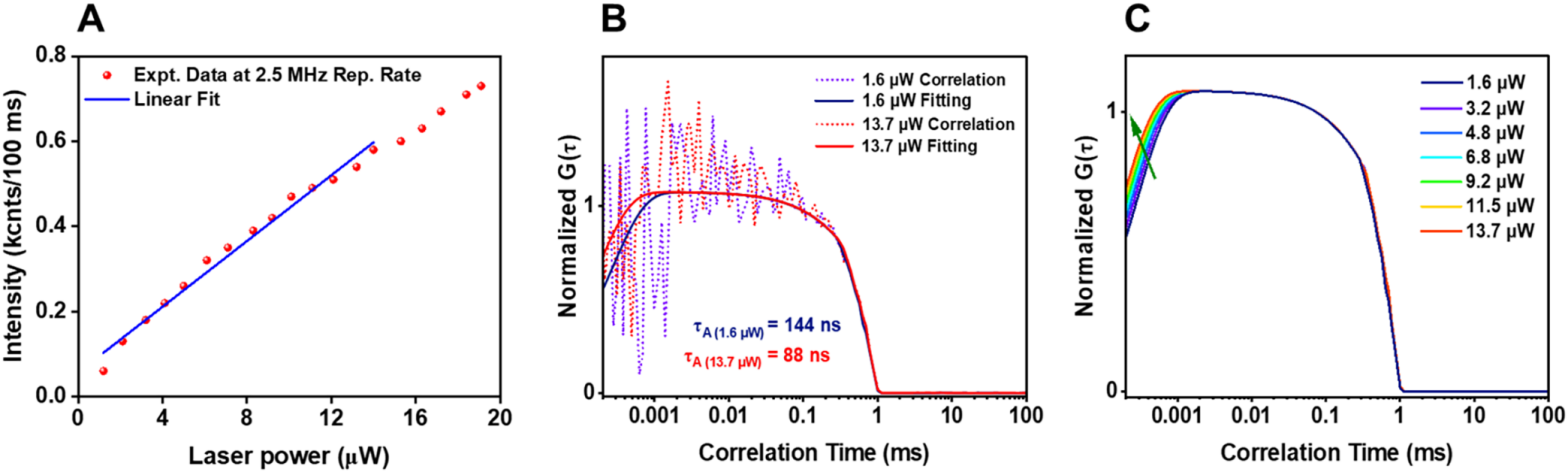
(A) Laser-power dependence over a power range of 1.2 to 19.1 μW. (B, C) Comparison of the numerical fits of the normalized FCS correlation of **Au**_**24**_ **(TBBM)**_**20**_ in toluene with increasing laser power from 1.6 to 13.7 μW. The arrow shows the trend in the numerical fits with increasing laser power. See Table S2 for the fitting parameters. Note: the lower threshold is selected to be above the background level during the FCS correlation measurements.

Antibunching experiments were performed at the lowest laser power (1.6 μW) that was consistent with obtaining a good signal-to-noise ratio, as lower power increases the depth of the antibunching dip by reducing the risk of photobleaching and probability of biexciton emission.^71,72^ We examined the antibunching dips in the two emission lifetime regimes (∼1 ns and ∼90 ns) seen for Au_24_(TBBM)_20_ in solution at the single molecule decay (see Figures 5A and S5A). A short emission lifetime is advantageous for SPE and for applications such as high-speed quantum communication and quantum key distribution, which require rapid single-photon generation. Generally speaking, a shorter exciton lifetime also reduces the chance of biexciton formation and therefore will enhance the purity of the single-photon source.^32,73^ Time-gated antibunching experiments were conducted as shown in Figure 5 with a data acquisition time of 30 min. All the antibunching data were fit using eq 1. Notably, a stronger antibunching dip is observed (Figure 5B) when time gating for the short lifetime component (0–10 ns). This corresponds to a single-photon purity, defined as the size of the antibunching dip relative to that of the sidebands, of 75%. In contrast, gating to select for the longer lifetime (30–400 ns) emission (Figure 5C) yielded a purity of approximately 69%. The standard deviation and average values over 5 FCS scans are shown in SI Figure S9, which shows a range of 72–78% SPE purity when gated for 0–10 ns (SI Figure S9A) and 65–71% SPE purity when gated for 30–400 ns (SI Figure S9B), respectively. For comparison and for validation purposes, single-particle FCS and antibunching data for the dye Coumarin 6 ^74^ in solution are shown in SI Figure S10A–D, which showed 85% SPE purity (see SI Figure S10D). Though the SPE characteristics of Au_24_(TBBM)_20_ NCs in the solution phase were confirmed through antibunching measurements, the relatively low purity of the signal may be attributed to their low PLQY in the visible (Table 1). Therefore, we investigated the NCs in the solid state, which is indeed more relevant for practical applications such as device fabrication.

**Figure 5:**
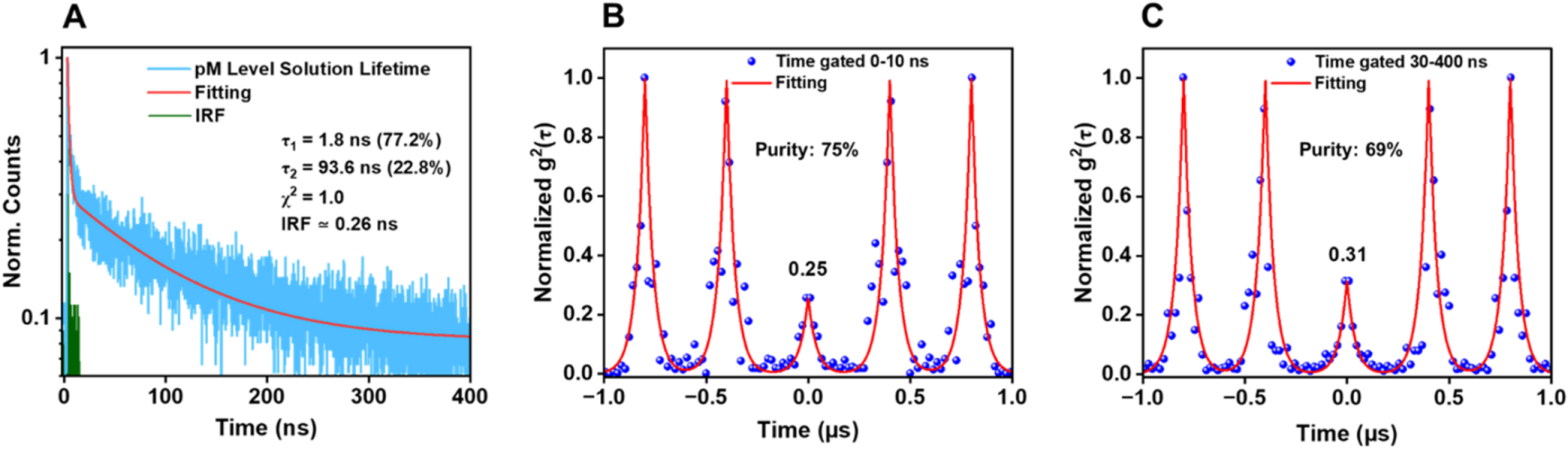
(A) PL lifetime of **Au**_**24**_ **(TBBM)**_**20**_ NC at ∼100 pM concentration in toluene, measured with a laser power of 5 μW. (B, C) Antibunching experiments in toluene solution using a 0–10 ns time gate and a 30–400 ns time gate, respectively. The excitation laser operates at 375 nm excitation with a repetition rate of 2.5 MHz and a power of 1.6 μW with a 680 ± 50 nm BP emission filter.

#### Solid-State SPE

Antibunching experiments were conducted in the solid state using fluorescence lifetime imaging microscopy (FLIM) to visualize the NCs. A representative FLIM image of single Au_24_(TBBM)_20_ NCs is shown in Figure 6A. The “donut-shaped” dipole emission pattern that is characteristic of single molecules was also observed for slightly defocused single NCs (Figure 6A, inset).^75,76^ In the solid state, NCs are more susceptible to photobleaching than in solution, as they are immobilized on the surface. To improve photostability, a protective PMMA coating was applied (see SI Section S3 for details) and a constant flow of helium gas was maintained over the film. A very low laser power of 0.6 μW was used to achieve stable emission as well. Notably, no visible blinking or bleaching was observed during the 6 min data acquisition period of our antibunching experiments (one representative time trace is shown in Figure 6B), confirming the photostability of the NCs throughout the measurements.

**Figure 6:**
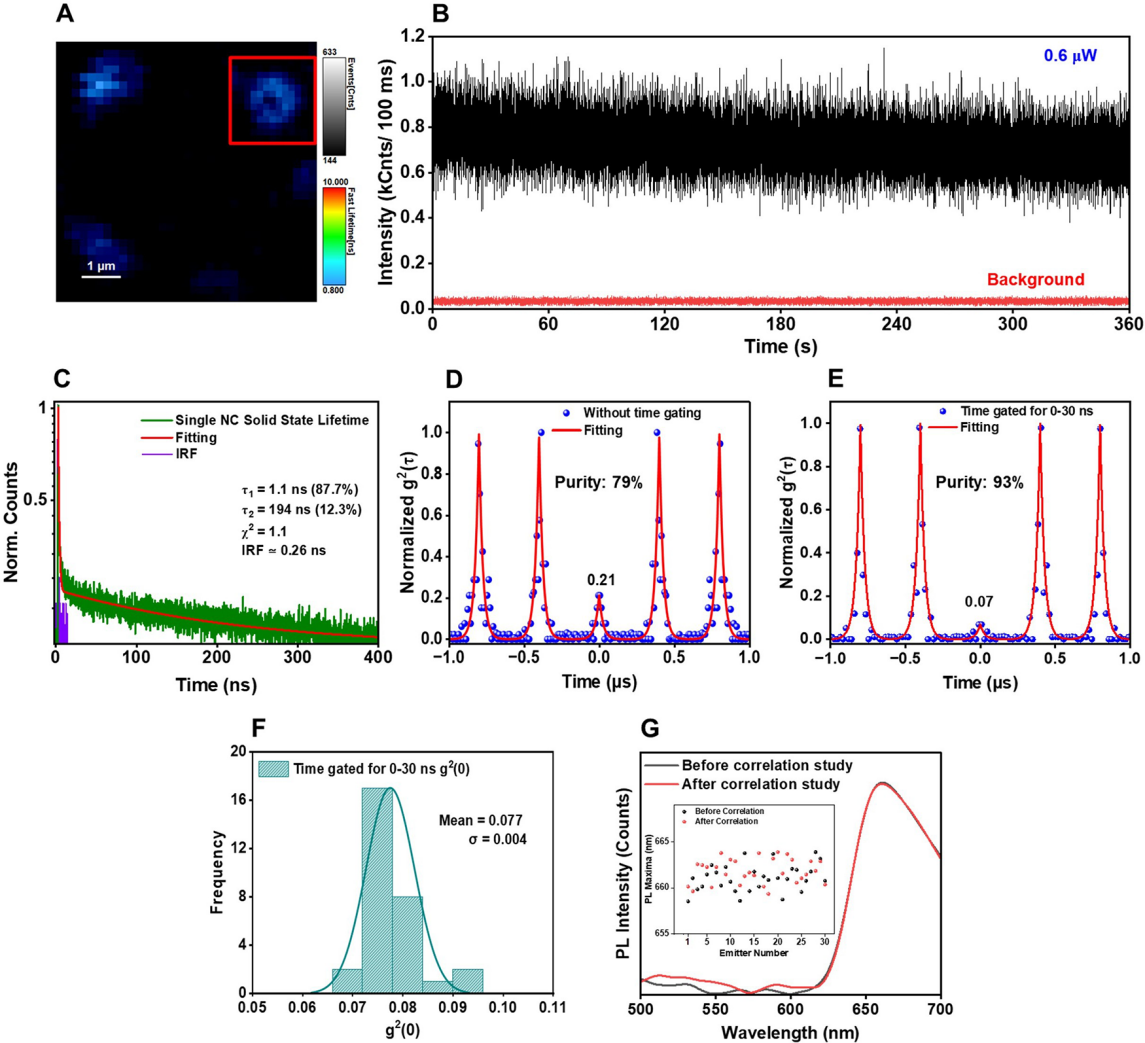
(A) A representative FLIM image with a color scale of **Au**_**24**_ **(TBBM)**_**20**_ NCs. In the inset: dipole emission pattern of a single NC. (B) The time-traces in 10 ms bins of a single **Au**_**24**_ **(TBBM)**_**20**_ NC collected during an antibunching measurement of 6 min with background counts at the bottom. (C) Single-particle PL lifetime of an **Au**_**24**_ **(TBBM)**_**20**_ NC in solid state, measured with a laser power of 5 μW. (D, E) Without and with time-gated (0–30 ns) antibunching experiments, respectively. (F) Histogram of *g* ^2^(0) values for 30 different **Au**_**24**_ **(TBBM)**_**20**_ NCs time gated for 0–30 ns. (G) The PL emission plot before and after an antibunching experiment for a single NC under consideration (inset: PL maxima check for 30 different emitters). For all the measurements, the NCs are in a PMMA matrix under helium gas purging conditions. The excitation laser operates at 375 nm with a 2.5 MHz repetition rate, 0.6 μW power, and a 680 ± 50 BP filter, except for the micro-PL experiments for which a 570 LP filter was used.

In the ensemble solid state (see Figure 3C) and single NC solid state lifetime analyses (see Figure 6C and SI Figure S5B), Au_24_(TBBM)_20_ NCs exhibit multicomponent decay dynamics, featuring a primarily short-lived (∼1 ns) component (see SI Figure S11). First, we performed the antibunching experiment in a solid state without any time gating, which resulted in almost 79% single-photon purity (Figure 6D). Subsequently, to evaluate the impact of the fast fluorescence on single-photon emission purity, we performed time-gated measurements for 0–30 ns in the solid state (Figure 6E). Interestingly, the fitting results indicate that gating for the short component yielded a single-photon purity of up to 93%.

We further analyzed 30 single NC emitters, selecting well-spaced objects from the FLIM images using time gating to select for the short lifetime emission (0–30 ns). The most probable *g* ^2^(0) value is obtained to be 0.077 and a very narrow range of values is measured (Figure 6F). Also, a histogram shows that on average the NCs emitted single photons with a SPE purity ranging between 90.8 and 93.0% (see SI Figure S12). These findings align with the high photon emission rate of Au_24_(TBBM)_20_ NCs in the solid state (see SI Figure S13).

To evaluate spectral stability of the NCs, their PL spectra was recorded before and after correlation experiments using a micro-PL setup incorporating a Gemini interferometer.^77^ At the single-particle level in the solid state, PL I is slightly red-shifted (∼13 nm) relative to the bulk-phase solid state measurements. Examining the emission spectra of 30 different emitters, no abrupt changes in the PL wavelength or intensity were observed before and after the antibunching experiments (one shown in Figure 6G and remainder in SI Figure S14), confirming the spectral stability of the Au_24_(TBBM)_20_ NCs (Figure 6G, inset). Lastly, the average photobleaching time for 30 NCs was measured, revealing a survival time of approximately 18–20 min without noticeable blinking under the experimental conditions used here (SI Figure S15). This precluded performing an “on” and “off” time analysis as is commonly done for systems that exhibit intermittent emission.

### Transient Absorption Studies in PMMA Film

Femtosecond transient absorption (fs-TA) measurements were conducted under the ambient conditions on the Au_24_(TBBM)_20_ NC film sample used in the bulk solid-state PL studies to better characterize the short-time dynamics that may not be observable in the fluorescence decay measurements. Upon photoexcitation at 380 nm, the film sample exhibits a broad excited-state absorption band (ESA, 450–750 nm) along with a ground-state bleach signal (GSB, ∼510 nm) but no clear evidence for stimulated emission was found (Figure 7A). Global analysis (Figure 7B) was performed to further study the exciton dynamics. As the longest time delay of the fs-TA apparatus used here is 7 ns, the fast decay time constants were fit by fixing the longest decay time constant to be >7 ns. Three-time constants, 0.8 ps, 22 ps, and 1.1 ns for PMMA film, were extracted (Figure 7B). The global fitting results were verified by kinetics fitting of selected wavelengths^78^ (500 and 650 nm) in PMMA film (see SI Figure S16B). The two fastest decay components (0.8 and 22 ps) in the solid state are attributed to excited-state electronic and vibrational relaxation processes, respectively.^79,80^ These dynamics are likely to reflect nonradiative processes that do not affect the emission rate as no corresponding IRF-limited decay was observed in the solid-state fluorescence lifetime measurements (see Figure 3C). We assign the 1.1 ns decay in PMMA to the depopulation of the S_1_ to the ground state, as this time scale closely matches the PL lifetimes obtained for bulk and single Au_24_(TBBM)_20_ NCs in PMMA (see Table 1, Figures 6C, and S11).

**Figure 7:**
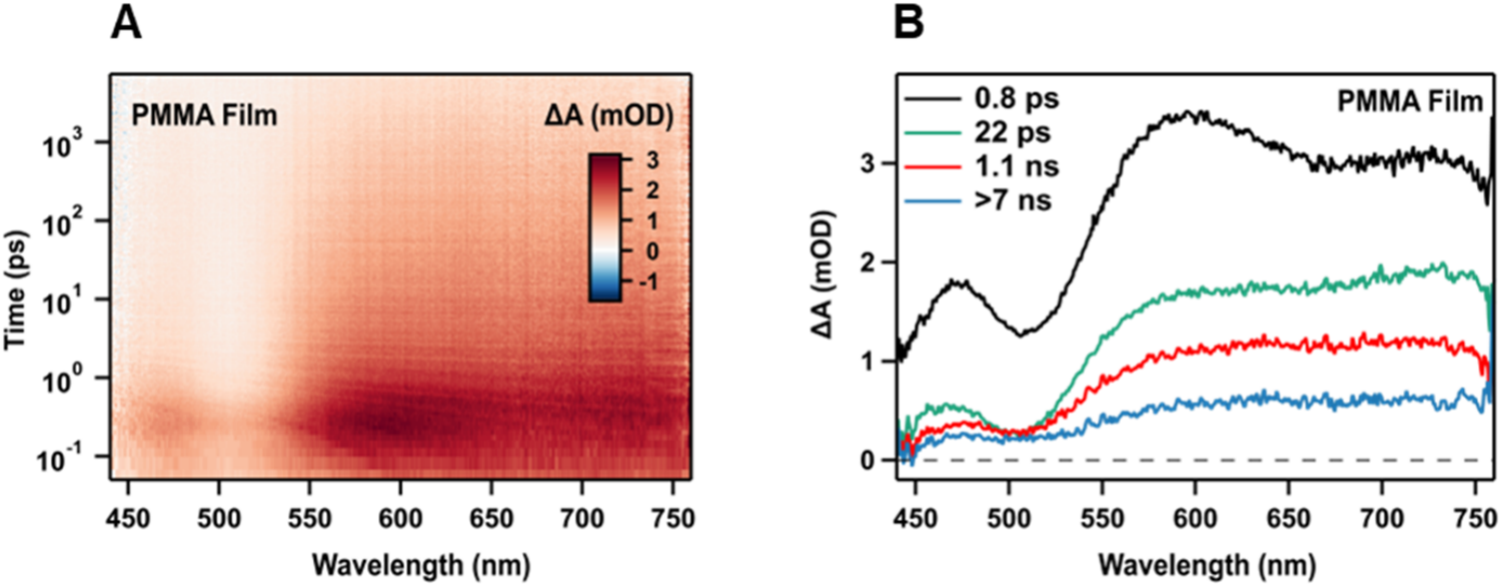
(A) Transient absorption spectra and (B) global analysis of **Au**_**24**_ **(TBBM)**_**20**_ NCs in PMMA film.

## Conclusions

In summary, this study systematically explores the SPE emission properties of Au_24_(TBBM)_20_ at room temperature in solution and the solid state. TA studies and time-gated fluorescence experiments indicate that a short lifetime in the nanosecond to subnanosecond range is beneficial for observing a larger photon antibunching dip (i.e., higher SPE purity). In solution, a single-photon purity of 75% is observed, while in the solid state at room temperature, short-time-gated measurements could achieve a much higher single-photon purity of 93%, with the emission being spectrally stable and with no noticeable blinking within our measurement time scale. The large improvement in the solid state is attributed to the much increased PLQY and having a predominantly fast decay process at the single-particle level. Additionally, lower laser power with PMMA polymer encapsulation and an inert atmosphere is found to be advantageous, reducing the risk of photobleaching of the quantum emitters. Overall, this work establishes a framework for studying SPE in atomically precise NCs at room temperature, highlighting the potential of such NCs as ideal SPE sources for quantum applications using gating electronics.

For future work, we plan to extend the SPE measurements to the near-infrared regime. Such emitters are critically needed in quantum telecommunication (Table S1). Atomically precise gold NCs with strong emission in the NIR have recently been reported,^11,37^ and their polarized emission may allow for high-efficiency collection of single photons. We believe that atomically precise NCs hold great potential for the development of near-infrared single photon emitters. The findings will contribute to the growing field of quantum nanophotonics, advancing the development of stable and efficient single-photon sources that are essential for quantum computing and cryptography applications.

## Methods and Experimental Section

### Synthesis of the Au_24_(TBBM)_20_ Nanocluster

The Au_24_(TBBM)_20_ NC was synthesized via a ligand exchange reaction using Au_25_(PET)_18_^–^ (PET: 2-phenylethanethiol) NC as the precursor.^54^ The precursor (∼2 mg) in toluene was reacted with 4-tert-butylbenzyl mercaptan (250 μL) at 80 °C under reflux for 2 h. After solvent removal, methanol washing, DCM extraction, and Thin Layer Chromatography purification (DCM:Pentane = 1:4) was performed to obtain the pure Au_24_(TBBM)_20_ NC. See the Supporting Information section for more details.

### Confocal Microscope for Single-Particle Studies

Single-particle confocal microscopy was performed using a 375 nm pulsed diode laser excitation with a 100× (NA 1.4) oil immersion objective on an inverted microscope. Emission was filtered (570 LP followed by 680 ± 50 nm BP), passed through an 80 μm pinhole, and split via a 50:50 beam splitter to two SPAD detectors in an HBT configuration, with photon timing recorded using PicoHarp 300/PHR 800 and analyzed in SymPhoTime 64 software. For single-particle PL measurements, a visible/NIR interferometer (GEMINI) with SPAD detection was employed. See the Supporting Information section for more details.

Other spectroscopic characterizations were performed using optical absorption, steady-state and time-resolved photoluminescence, as well as transient absorption techniques. Additional analyses were conducted by using transmission electron microscopy. See the Supporting Information for more details.

## Supplementary Material



## Notes

Detailed descriptions of the Au_24_(TBBM)_20_ nanocluster synthesis; preparation procedures for optical and electron microscopy samples; HAADF-STEM images of Au_24_(TBBM)_20_ nanoclusters and size distribution analysis; bootstrap analysis of bulk TCSPC fits; power dependence of solution phase and solid state single-emitter TCSPC lifetime decays and fits for Au_24_(TBBM)_20_; FCS data and fits for Au_24_(TBBM)_20_ showing power dependence and effects of additional triplet term in FCS analysis; measurements of FCS and antibunching for a laser dye (Coumarin 6) in toluene; measurements of distributions of single-emitter lifetimes, photon purity, emission intensity, and emission spectra; single-emitter intermittency and single step bleaching behavior of Au_24_(TBBM)_20_ in PMMA; femtosecond transient absorption kinetics and fits of Au_24_(TBBM)_20_ in PMMA; compilation of recent literature results on high-purity nanoscale single photon sources for comparison to results for Au_24_(TBBM)_20_ (PDF)
