# Supplementary material for: High-Purity Quantum Emission from an Au24(S-CH2Ph‑ t Bu)20 Nanocluster at Room Temperature
Source: ACS Nano. 2026 May 14;20(20):14879–89. doi: 10.1021/acsnano.6c05162 (PMC13218048; doi:10.1021/acsnano.6c05162)
Supplement: Supplementary file 1 [file nn6c05162_si_001.pdf]

Supporting Information:

High Purity Quantum Emission from an $Au_{24}(S\text{-}CH_2Ph\text{-}^tBu)_{20}$ Nanocluster at Room Temperature

Abhrojyoti Mazumder[1], Ece Gunay[2], Guiying He[1], Zhongyu Liu[1], Sebastian Calderon[2], Lianshun Luo[1], Yitong Wang[1], Mircea Cotlet[3], Elizabeth C. Dickey[2]*, Rongchao Jin[1]* and Linda A. Peteanu[1]*

[1] Department of Chemistry, Carnegie Mellon University, Pittsburgh, PA 15213 USA

[2] Department of Materials Science and Engineering, Carnegie Mellon University, Pittsburgh, PA 15213 USA

[3] Center for Functional Nanomaterials, Brookhaven National Laboratory, Upton, NY 11973, USA

Corresponding authors: ecdickey@andrew.cmu.edu, rongchao@andrew.cmu.edu, peteanu@andrew.cmu.edu

Experimental:

1. Materials and Reagents

Tetrachloroauric (III) acid ($HAuCl_4 \cdot 3H_2O$, 99.999% metal basis, Aldrich), tetraoctylammonium bromide (TOAB, ≥98%, Aldrich), 2-phenylethanethiol (PET, 98%, Aldrich), 4-tert-butylbenzyl mercaptan (TBBM, 98%, Aldrich), sodium borohydride ($NaBH_4$, Aldrich) and Polymethyl methacrylate (PMMA, Aldrich) were used as received without further purification. The solvents included methanol (HPLC grade, ≥99.9%, Aldrich), dichloromethane (DCM, ACS reagent, ≥99.5%, Aldrich), pentane (ACS grade, ≥99.9%, Aldrich), acetone (ACS grade, ≥99.5%, Aldrich), acetonitrile (HPLC grade, ≥99.9%, Aldrich) and toluene (HPLC grade, ≥99.9%, Aldrich). Microscope cover glass (22×22 mm, Fisherbrand). Nanopure water from Barnstead NANOpure Diamond system. Thin-layer chromatography (TLC) plates (250 µm silica gel) from iChromatography.

2. Synthesis of the $Au_{24}(TBBM)_{20}$ Nanocluster

$Au_{24}(TBBM)_{20}$ was synthesized via a previously reported ligand exchange method.[s1] Here, the $Au_{25}(PET)_{18}^-$ nanocluster (NC) served as the precursor for the ligand exchange. Briefly, the $Au_{25}(PET)_{18}^-$ TOA^+ (TOA^+: tetraoctylammonium) NC was first synthesized following established procedures.[s2,s3] Approximately 2 mg of the purified $Au_{25}(PET)_{18}^-$ NC was then dissolved in 1 mL of toluene in a 10 mL round-bottom flask equipped with a magnetic stir bar. Next, 0.25 mL of $HSCH_2Ph\text{-}^tBu$ thiol was added to the solution. The mixture was stirred at 60 rpm for 2 hours at 80 °C under reflux conditions. Afterward, the toluene was evaporated under vacuum, and the sample was washed twice with methanol to remove excess thiol. Then it was extracted with DCM. Finally, thin-layer chromatography (TLC) was performed using a dichloromethane:pentane (1:4 v/v) solvent mixture to obtain the purified $Au_{24}(TBBM)_{20}$ NC.

3. Preparation of $Au_{24}(TBBM)_{20}$/ PMMA Solid Thin Films

A. For bulk studies**:** A uniform solid thin film of the NC/PMMA sample was prepared by drop casting. To prepare the PMMA stock solution, 40 mg of PMMA was dissolved in 1 mL of DCM. Separately, a stock solution of $Au_{24}(TBBM)_{20}$ NCs was prepared by dissolving 1 mg of the NCs in 1 mL of DCM. Subsequently, 10 μL of the PMMA solution was mixed with 20 μL of the NC solution to form an ink-like NC/PMMA mixture. Finally, 25 μL of the mixture was drop cast onto a quartz plate and air dried at room temperature for an hour.

B. For single NC studies: First, a picomolar level $Au_{24}(TBBM)_{20}$ NC solution was prepared in degassed DCM. Next, the NC solution was combined with a polymer matrix (a 2 wt% PMMA solution in degassed DCM). The resulting solution was spin coated at 1500 rpm for 120 seconds (Laurell WS-650MZ-23NPP spin coater) onto a coverslip that was first washed with isopropanol and then dried with N_2. The resulting sample slide was dried for an hour under N_2 gas at room temperature.

4. Steady-State UV-Vis Measurements

The UV–Vis spectra for the $Au_{24}(TBBM)_{20}$ NC was obtained using a UV-3600 Plus spectrophotometer (Shimadzu) with a wavelength range of 185-3300 nm.

5. Steady-State and Time-Resolved Photoluminescence Measurements

Steady-state photoluminescence spectra were recorded using an FLS-1000 spectrofluorimeter (Edinburgh). Photoluminescence lifetimes were determined through time-correlated single photon counting (TCSPC) technique using a pulsed laser having a 25 ps pulse width.

6. Transient Absorption Measurements

For femtosecond transient absorption measurements, a commercially available (Spectra-Physics) Ti:Sapphire laser system (1 kHz) equipped with an optical parametric amplifier is used to generate pump pulses (380 nm) with a pulse width of ~100 fs. Supercontinuum probe light was generated by focusing the 800 nm fundamental into a sapphire plate. The delay between pump and probe pulse was controlled by a mechanical delay stage (up to 7 ns). The transient absorption spectra were analyzed using the publicly available program Glotaran based on the statistical fitting package TIMP. A sequential model was adopted to give the evolution associated spectra (EAS).[s4,s5]

7. Transmission Electron Microscopy Measurements

Transmission electron microscopy (TEM) samples were prepared using a drop-casting method onto a Cu grid coated with an ultrathin lacey carbon support layer. High-angle annular dark-field scanning transmission electron microscopy (HAADF-STEM) imaging was conducted on an aberration-corrected Thermo Fisher Themis microscope operating at 80 kV. Imaging was carried out using a collection angle of 46–200 mrad, with a convergence angle of 30 mrad and a beam current of 17 pA.

8. Confocal Microscopy Measurements

For our single-particle confocal microscopy the excitation was provided by a 375 nm pulsed diode laser (Picoquant). A 1.4 NA 100× oil immersion objective (Olympus UPlanSAPO 100×1.4 oil) was mounted on an inverted microscope (Olympus IX-71). Emission was directed through an 80 μm pinhole to a 50:50 beam splitter (Semrock), which divided the signal between two single-photon detectors (Micro Photon Devices

PDM50) in a Hanbury Brown and Twis (HBT) configuration. The apparatus incorporates the following filters: a 680 ± 50 nm bandpass, a 390 nm dichroic, 400 nm and 570 nm long pass filters (all from Thorlabs). Photon time-tagging was performed using the PicoHarp 300 and PHR 800 electronics (Picoquant). Data collection and analysis—including intensity time traces, lifetime measurements, antibunching, and fluorescence correlation—were conducted using the SymPhoTime software package (Picoquant). Excitation powers ranged from 0.6 to 19.1 µW, as measured with a PM100D power meter and an S120VC sensor from Thorlabs before the beam entered the microscope. For the single particle PL measurements, a visible/NIR transmitting interferometer (GEMINI, Nireos Inc.) was mounted on the right-side port of the microscope and a SPAD (Micro Photon Devices PDM50) was used to detect the emitted light.

Supporting Figures:

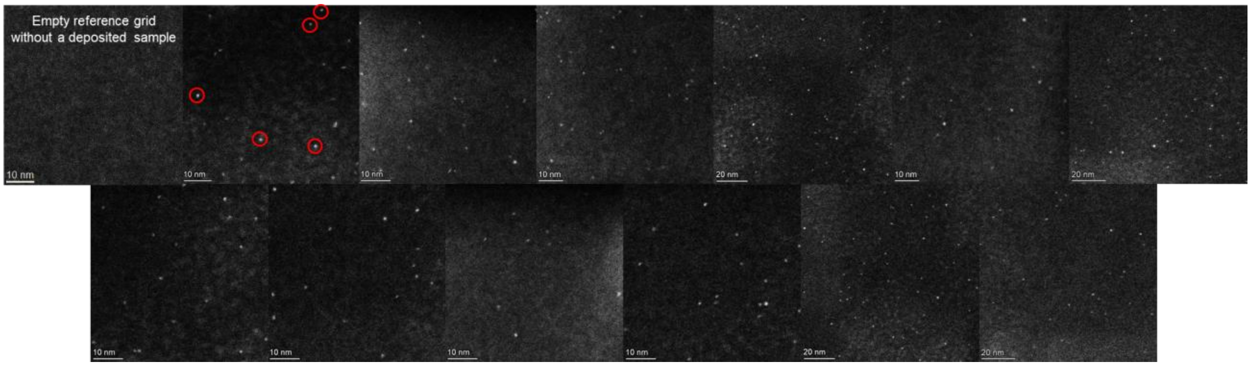

Figure S1. HAADF-STEM image acquired from an empty reference grid, illustrating the background contrast in the absence of a specimen, and representative images from multiple regions used for particle size analysis. In total, ~200 nanoclusters at a pM-level concentration were analyzed. Red circles are shown in the selected panel to highlight example particles.

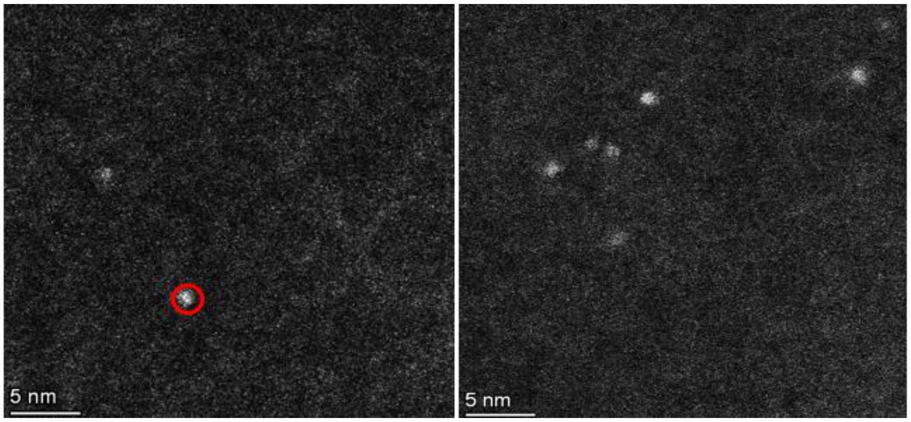

Figure S2. Representative high-magnification HAADF images illustrating the typical particle density observed at ~100 pM-level concentration. These images demonstrate the limited number of NCs visible per field of view at higher magnification. Due to the dilute conditions, multiple regions at lower magnification were imaged and combined for statistical analysis.

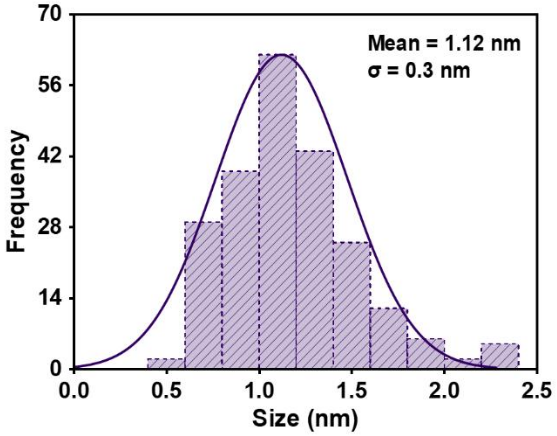

Figure S3. Size distribution histogram collected from HAADF-STEM image of **$Au_{24}(TBBM)_{20}$** NCs.

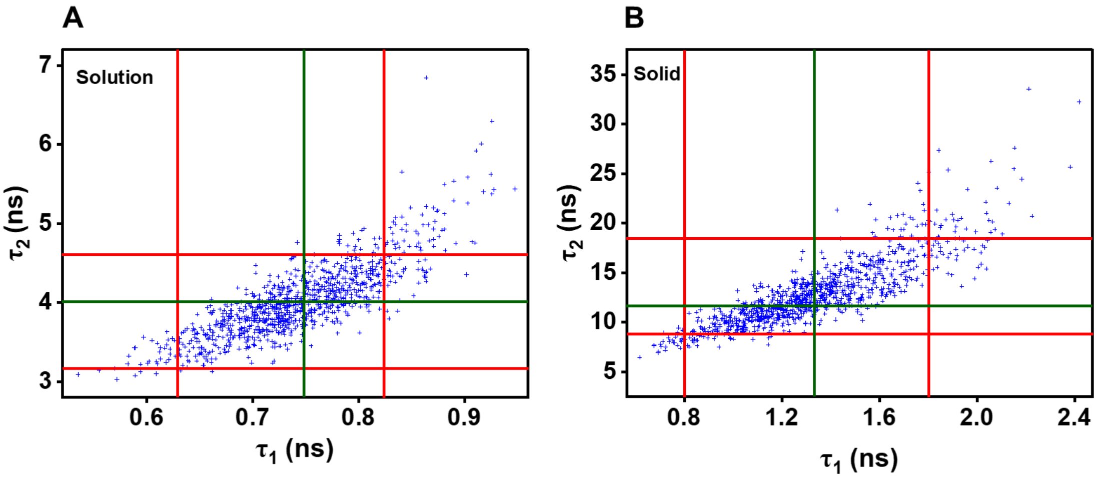

Figure S4. Early time bootstrap analysis of the bulk-phase TCSPC decay fitting for **$Au_{24}(TBBM)_{20}$** NCs in (A) Toluene solution and (B) PMMA rigid matrix at room temperature. The error ranges are denoted with red lines.

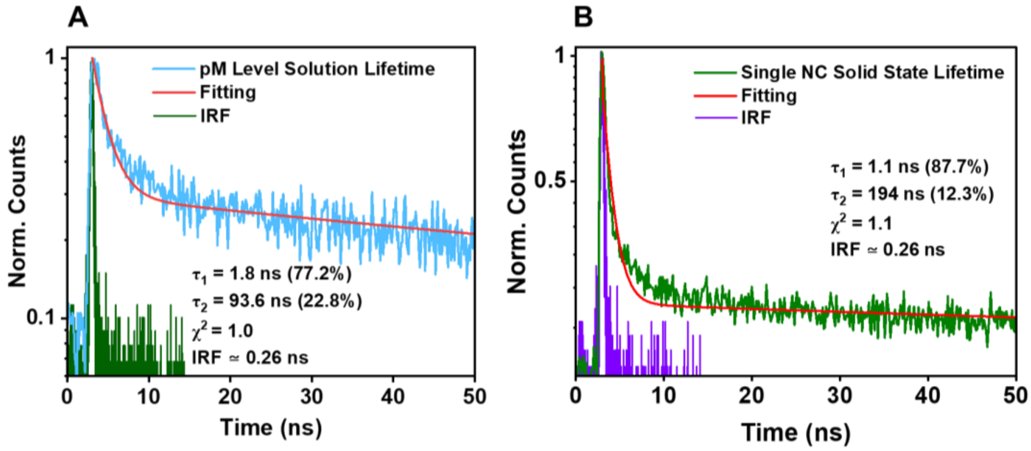

Figure S5. (A) Early time decay with IRF of the PL lifetime of **$Au_{24}(TBBM)_{20}$** NC at ~100 pM concentrations in toluene. (B) Early time decay with IRF of the single particle PL lifetime of **$Au_{24}(TBBM)_{20}$** NC in solid-state (embedded in a PMMA matrix). Measured with a laser power of 5 μW.

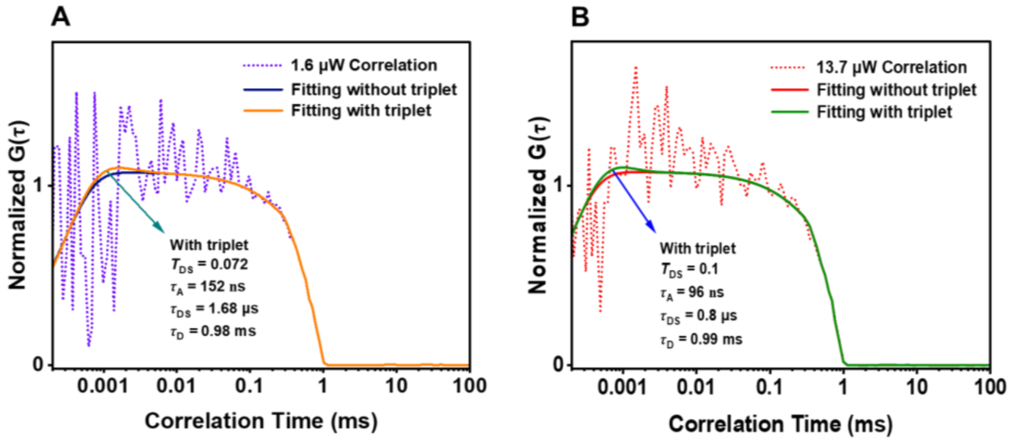

Figure S6. FCS data fitting comparison in toluene with and without the triplet term at (A) 1.6 μW and (B) 13.7 μW in toluene. The excitation laser operates at 375 nm with a 2.5 MHz repetition rate, 1.6 μW power, and a 680 ± 50 nm BP filter was used. Scanned for 5 min.

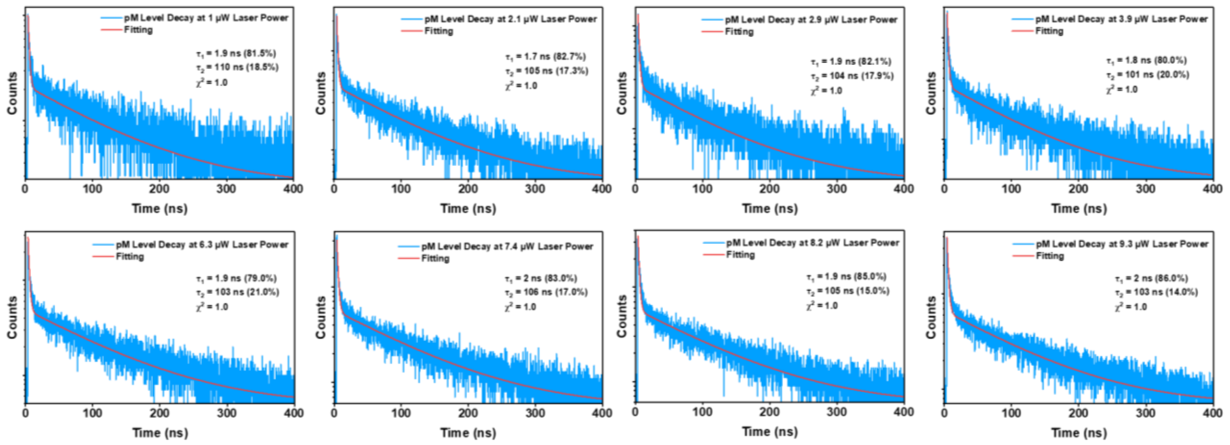

Figure S7. Laser power dependent PL lifetime fits of **Au_{24}(TBBM)$_{20}$** NC at ~100 pM concentration in toluene, over a power range of 1.0 to 9.3 μW (Note: 5 μW lifetime fit is shown in the manuscript Fig. 5A).

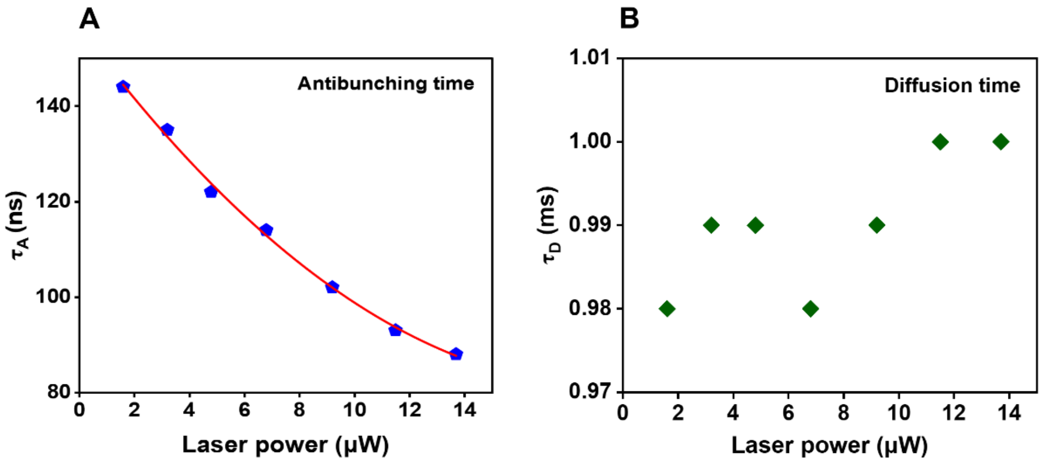

Figure S8. Power-dependent FCS studies in toluene: (A) antibunching time and (B) diffusion time.

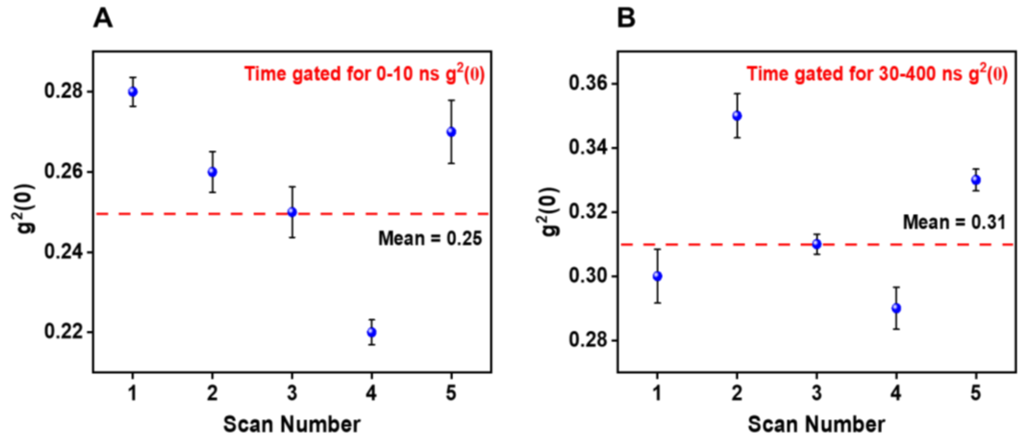

Figure S9. Mean $g^2(0)$ values obtained from time gated antibunching studies in solution (error bars in the Y-axis, obtained during the data fitting).

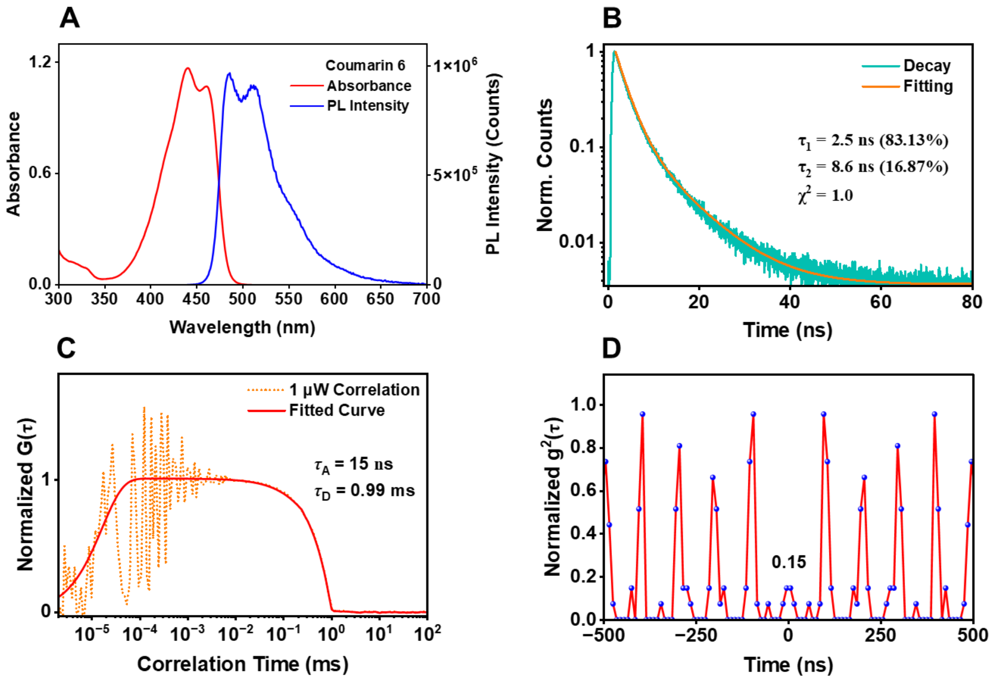

Figure S10. Coumarin 6: (A) Bulk absorption and PL profile. (B) TCSPC decay profile. (C-D) FCS and antibunching measurements respectively of a ~10 pM level Coumarin 6 solution in toluene under 375 nm excitation at a 10 MHz repetition rate, with a laser power of 1 μW, scanned for 5 min.

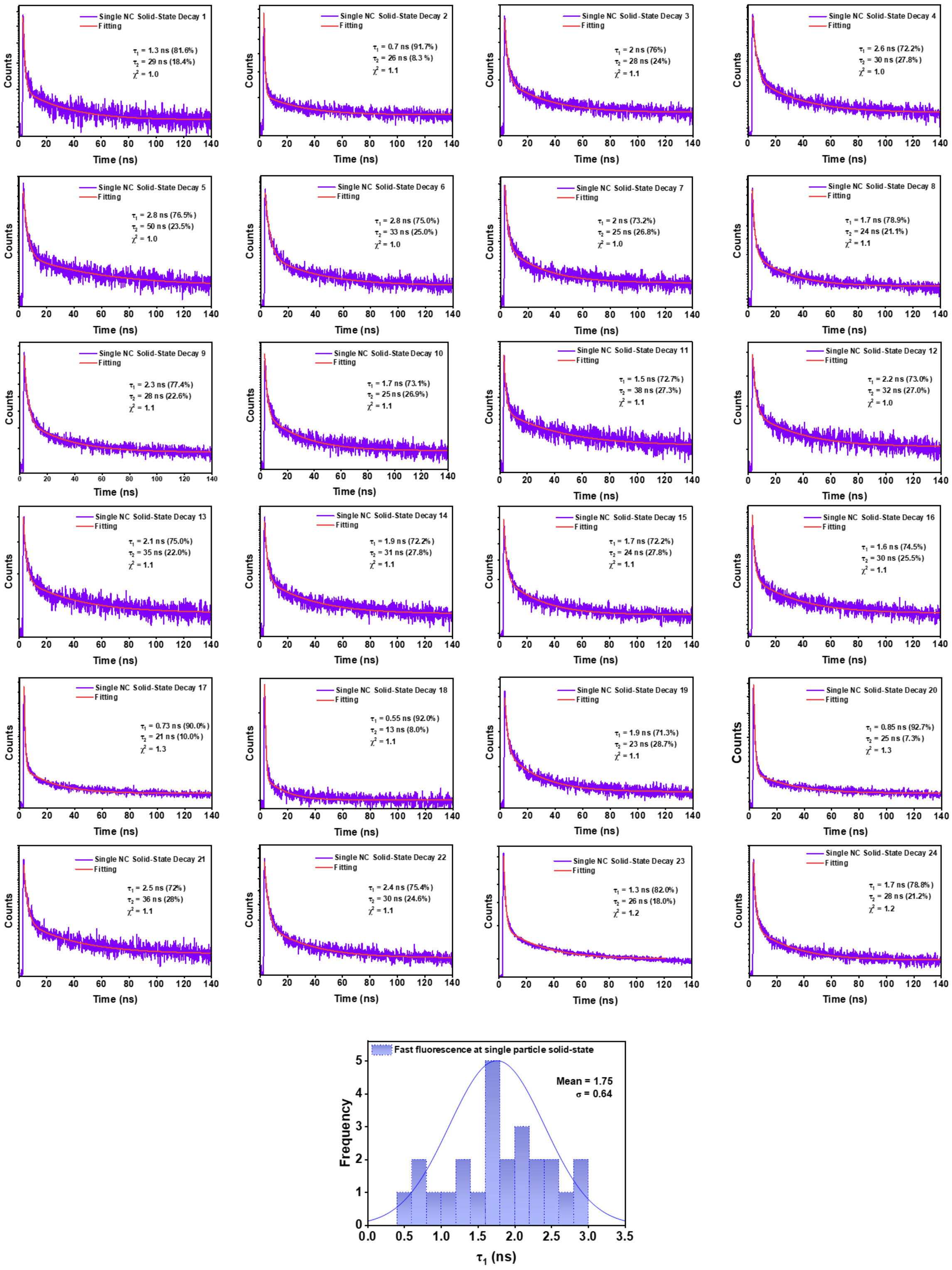

Figure S11. Single **$Au_{24}(TBBM)_{20}$** NC decay profiles for 24 different NCs embedded in PMMA matrix under helium gas atmosphere at room temperature, resulting in an average fast fluorescence decay (τ_1) of 1.75 ns.

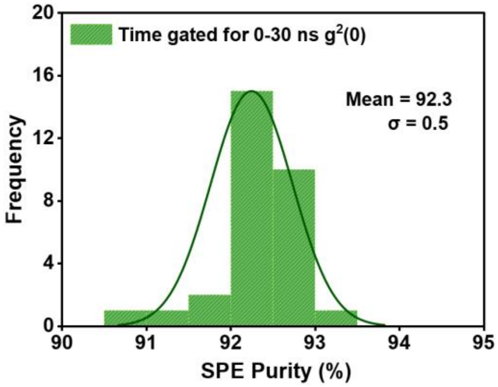

Figure S12. Emitted single photon purity check for 30 emitters embedded into a PMMA matrix under helium gas atmosphere.

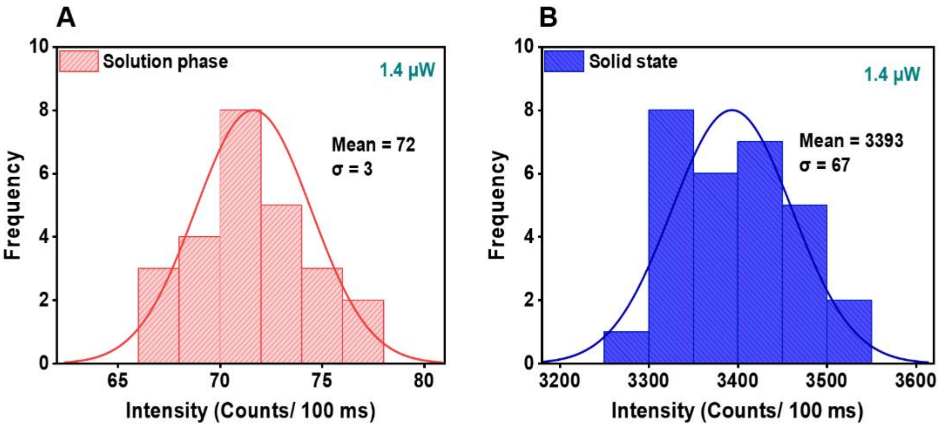

Figure S13. Comparison of the single-particle emission intensity (averaged over 30 particles) between (A) picomolar-level toluene solution and (B) PMMA-embedded NCs, both at 1.4 μW. Note: The background is ~20-30 counts/100 ms.

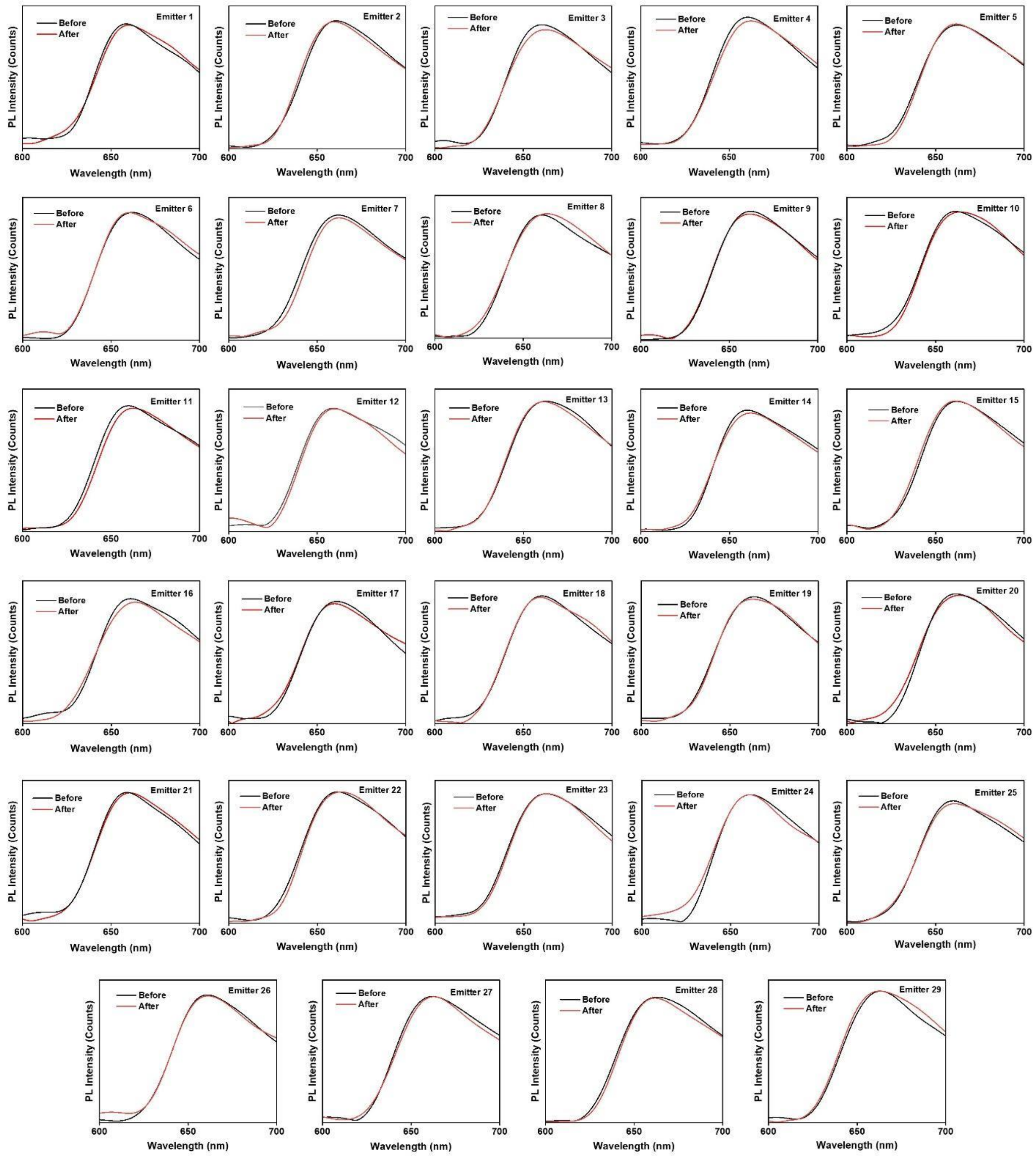

Figure S14. Single **Au_{24}(TBBM)$_{20}$** NC PL spectra before and after the antibunching experiments for 29 different NCs embedded into a PMMA matrix under helium gas atmosphere at room temperature.

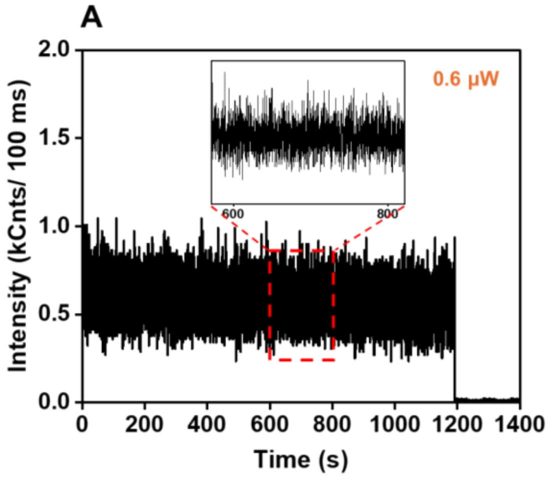

Figure S15. Single step photobleaching time analysis for 30 isolated **$Au_{24}(TBBM)_{20}$** NCs embedded in a PMMA matrix under helium gas atmosphere at room temperature.

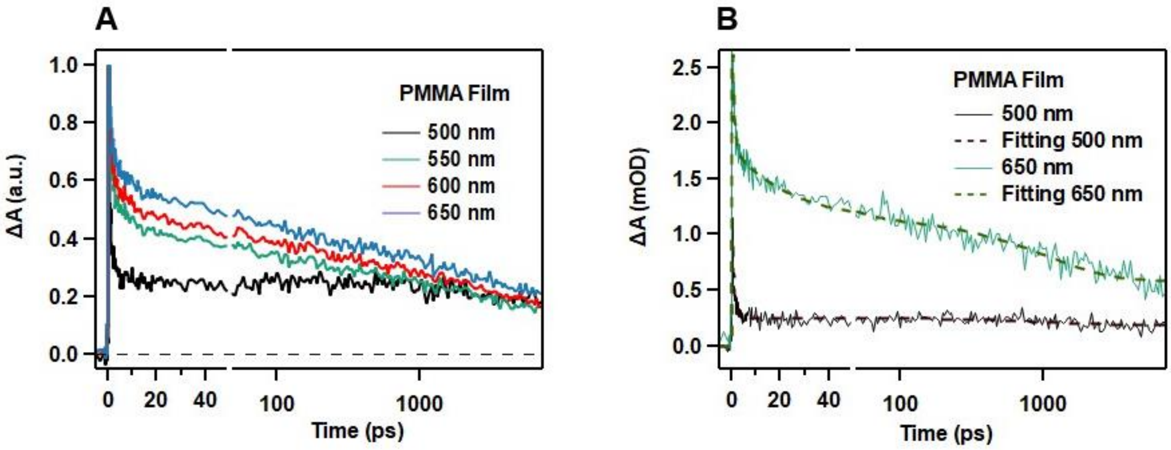

Figure S16. Transient absorption data analysis of **$Au_{24}(TBBM)_{20}$** NCs in PMMA film: (A) Kinetics at single wavelengths extracted from fs-TA data. (B) Kinetics fitting results at selected wavelengths. Derived from the global analysis of **$Au_{24}(TBBM)_{20}$** NCs PMMA film (see Fig. 7B).

Table S1. Summary of Selected High-Purity Nanoscale Single-Photon Sources with Potential for Quantum Technology

Source	Peak Emission (λ_{max}) Wavelength (nm)	Lifetime at Room Temperature (ns)	$g^2(0)$	Operational Temperature	Reference
Diamond (NV^-/NV^0)	~637 (NV^-), ~575 (NV^0)	~10–20	<0.1	RT	s6

Hexagonal boron nitride (C-related and B-center related defects)	~430-580 (defect dependent)	~1–6	As low as ≈ 0.015 (on avg. <0.1)	RT	s7,s8
GeV color center in diamond	602 (narrow ZPL)	~1.4	<0.1	RT	s9
SiC Based (Intrinsic defects in 3C-SiC epitaxial film and single divacancy spin defects in 4H-SiC)	~850–1,300 (incl. telecom)	~1–20 (defect-, polytype-dependent)	<0.5 (device-dependent)	RT to Cryogenic (4K)	s10,s11
III-N QDs: InGaN/GaN (nanowires / m-plane / QDs)	~400–560	~0.35–1.3	≈ 0.043–0.47	Cryogenic (9K) to RT	s12-14
Perovskite QDs ($CsPbI_3$ / $CsPbBr_3$)	~520–700	Not explicitly reported	≈ 0.02–0.1 (without cavities)	RT	s15
Point defects in GaN	Telecom	0.67	<0.1	RT	s16
$CsPbI_3$ in optical microcavity	677	12.7	0.06	RT	s17
Native defects in hBN coupled to plasmonic nano-resonators (gold + alumina spacer)	~585–675	~0.480 (with alumina spacer), <0.5 avg.	$g^2(0)$ = 0.29–0.48; (~29% of emitters < 0.5)	RT	s18
PbS/CdS colloidal QD (embedded in a nanoparticle-on-mirror plasmonic nanogap cavity)	~1,550 (telecom C-band) or 1350 nm (O-band)	~0.208-0.065 (cavity coupled)	0.27-0.29	RT	s19
Graphene QD	~650	5.37	<0.1	RT	s20

InP/ZnSe/ZnS colloidal quantum dots (heavy-metal-free)	~613	~15 (without plasmonic antenna) ~125-0.250 (using plasmonic nanoantenna)	0.077–0.086	4 K (Cryogenic)	s21
Perylene bisimide (PBI) dye molecules adsorbed on CdSe/ZnS quantum dots	~620	~3.5 (avg.)	<0.1	RT	s22
Dihydrolipoic acid protected Ag_{29} and $Ag_{28}Au$ nanoclusters	~657	~3-2.5 (single particle)	Not explicitly reported	RT	s23
**This work**	~650 (solid-state)	~10-25 (on avg. solid-state single particle)	~0.07 (time-gated for fast emission lifetime ~1 ns)	RT	

FCS data fitting parameters:

For FCS data fitting, we utilized the three-dimensional free diffusion model (refer to eq. 2 in the main text). During the fitting process, we set the N_{emi} parameter to unity, assuming a single emitting species per molecule, resulting in N being approximately 1 in all cases. We assigned a value of $\omega = 5$, which was suitable for our confocal instrument. To extract various photophysical parameters, we fitted the experimental data over a time range from 1 ns to 100 ms.

Table S2. Results from Fitting the Power Dependent FCS Curves for $Au_{24}(TBBM)_{20}$ in Toluene Solution at Room Temperature

Laser Power (µW) [a]	τ_A (ns)	τ_D (ms)
1.6	144	0.98
3.2	135	0.99
4.8	122	0.99
6.8	114	0.98
9.2	102	0.99
11.5	93	1.00
13.7	88	1.00

[a]375 nm excitation with a repetition rate of 2.5 MHz.

References:

(s1) Das, A.; Li, T.; Li, G.; Nobusada, K.; Zeng, C.; Rosi, N. L.; Jin, R. Crystal Structure and Electronic Properties of a Thiolate-Protected Au_{24} Nanocluster. *Nanoscale* **2014**, *6* (12), 6458–6462. https://doi.org/10.1039/c4nr01350f.

(s2) Liu, Z.; Zhou, M.; Luo, L.; Wang, Y.; Kahng, E.; Jin, R. Elucidating the Near-Infrared Photoluminescence Mechanism of Homometal and Doped $M_{25}(SR)_{18}$ Nanoclusters. *J Am Chem Soc* **2023**, *145* (36), 19969–19981. https://doi.org/10.1021/jacs.3c06543.

(s3) Wu, Z.; Suhan, J.; Jin, R. One-Pot Synthesis of Atomically Monodisperse, Thiol-Functionalized Au_{25} Nanoclusters. *J Mater Chem* **2009**, *19* (5), 622–626. https://doi.org/10.1039/b815983a.

(s4) van Stokkum, I. H. M.; Larsen, D. S.; van Grondelle, R. Global and Target Analysis of Time-Resolved Spectra. *Biochim. Biophys. Acta, Bioenerg.* **2004**, *1657*, 82–104. https://doi.org/10.1016/j.bbabio.2004.04.011.

(s5) Snellenburg, J. J.; Laptenok, S. P.; Seger, R.; Mullen, K. M.; van Stokkum, I. H. M. *Glotaran: A Java-Based Graphical User Interface for the R Package TIMP. J. Stat. Softw.* **2012**, *49* (3), 1–22. https://doi.org/10.18637/jss.v049.i03.

(s6) Berthel, M.; Mollet, O.; Dantelle, G.; Gacoin, T.; Huant, S.; Drezet, A. Photophysics of Single Nitrogen-Vacancy Centers in Diamond Nanocrystals. *Phys. Rev. B* **2015**, *91* (3), 035308. https://doi.org/10.1103/PhysRevB.91.035308.

(s7) Chatterjee, A.; Biswas, A.; Fuhr, A. S.; Terlier, T.; Sumpter, B. G.; Ajayan, P. M.; Aharonovich, I.; Huang, S. Room-Temperature High-Purity Single-Photon Emission from Carbon-Doped Boron Nitride Thin Films. *Sci. Adv.* **2025**, *11* (25), eadv2899. https://doi.org/10.1126/sciadv.adv2899.

(s8) Yamamura, K.; Coste, N.; Zeng, H. Z. J.; Toth, M.; Kianinia, M.; Aharonovich, I. Quantum Efficiency of the B-Center in Hexagonal Boron Nitride. *Nanophotonics* **2025**, *14* (11), 1715–1720. https://doi.org/10.1515/nanoph-2024-0412.

(s9) Iwasaki, T.; Ishibashi, F.; Miyamoto, Y.; Doi, Y.; Kobayashi, S.; Miyazaki, T.; Tahara, K.; Jahnke, K. D.; Rogers, L. J.; Naydenov, B.; Jelezko, F.; Yamasaki, S.; Nagamachi, S.; Inubushi, T.; Mizuochi, N.; Hatano, M. Germanium-Vacancy Single Color Centers in Diamond. *Sci. Rep.* **2015**, *5*, 12882. https://doi.org/10.1038/srep12882.

(s10) Wang, J.; Zhou, Y.; Wang, Z.; Rasmita, A.; Yang, J.; Li, X.; von Bardeleben, H. J.; Gao, W. Bright Room Temperature Single Photon Source at Telecom Range in Cubic Silicon Carbide. *Nat. Commun.* **2018**, *9*, 4106. https://doi.org/10.1038/s41467-018-06605-3.

(s11) Li, Q.; Wang, J.-F.; Yan, F.-F.; Zhou, J.-Y.; Wang, H.-F.; Liu, H.; Guo, L.-P.; Zhou, X.; Gali, A.; Liu, Z.-H.; Wang, Z.-Q.; Sun, K.; Guo, G.-P.; Tang, J.-S.; Li, H.; You, L.-X.; Xu, J.-S.; Li, C.-F.; Guo, G.-C. Room-Temperature Coherent Manipulation of Single-Spin Qubits in Silicon Carbide with a High Readout Contrast. *Natl. Sci. Rev.* **2022**, *9* (5), nwab122. https://doi.org/10.1093/nsr/nwab122.

(s12) Holmes, M. J.; Zhu, T.; Massabuau, F. C.-P.; Jarman, J.; Oliver, R. A.; Arakawa, Y. Pure Single Photon Emission from an InGaN/GaN Quantum Dot. *Appl. Phys. Lett.* **2021**, *118* (21), 211108. https://doi.org/10.1063/5.0052543.

(s13) Wang, T.; Puchtler, T. J.; Zhu, T.; Jarman, J. C.; Nuttall, L. P.; Oliver, R. A.; Taylor, R. A. Polarisation-Controlled Single Photon Emission at High Temperatures from InGaN Quantum Dots. *Nanoscale* **2017**, *9* (27), 9421–9427. https://doi.org/10.1039/C7NR03391E.

(s14) Saha, P. K.; Rana, K. S.; Thakur, N.; Parvez, B.; Bhat, S. A.; Ganguly, S.; Saha, D. Room Temperature Single-Photon Emission from InGaN Quantum Dot Ordered Arrays in GaN Nanoneedles. *Appl. Phys. Lett.* **2022**, *121* (21), 211101. https://doi.org/10.1063/5.0111026.

(s15) Zhu, C.; Marczak, M.; Feld, L.; Boehme, S. C.; Bernasconi, C.; Moskalenko, A.; Cherniukh, I.; Dirin, D.; Bodnarchuk, M. I.; Kovalenko, M. V.; Rainò, G. Room-Temperature, Highly Pure Single-Photon Sources from All-Inorganic Lead Halide Perovskite Quantum Dots. *Nano Lett.* **2022**, *22* (9), 3751–3760. https://doi.org/10.1021/acs.nanolett.2c00756.

(s16) Meunier, M.; Eng, J. J. H.; Mu, Z.; Chenot, S.; Brändli, V.; de Mierry, P.; Gao, W.; Zúñiga-Pérez, J. Telecom Single-Photon Emitters in GaN Operating at Room Temperature: Embedment into Bullseye Antennas. *Nanophotonics* **2023**, *12* (8), 1405–1419. https://doi.org/10.1515/nanoph-2022-0659.

(s17) Farrow, T.; Dhawan, A. R.; Marshall, A. R.; Ghorbal, A.; Son, W.; Snaith, H. J.; Smith, J. M.; Taylor, R. A. Ultranarrow Line Width Room-Temperature Single-Photon Source from Perovskite Quantum Dot Embedded in Optical Microcavity. *Nano Lett.* **2023**, *23* (23), 10667–10673. https://doi.org/10.1021/acs.nanolett.3c02058.

(s18) Sakib, M. A.; Triplett, B.; Harris, W.; Hussain, N.; Senichev, A.; Momenzadeh, M.; Bocanegra, J.; Vabishchevich, P.; Wu, R.; Boltasseva, A.; Shalaev, V. M.; Shcherbakov, M. R. Purcell-Induced Bright Single Photon Emitters in Hexagonal Boron Nitride. *Nano Lett.* **2024**, *24* (40), 12390–12397. https://doi.org/10.1021/acs.nanolett.4c02581.

(s19) Zhang, S.; Traverso, A. J.; Dolgopolova, E. A.; Singh, A.; Kishida, H.; Livshits, M. Y.; Sheehan, C. J.; Bowes, E. G.; Li, C.; Hollingsworth, J. A.; Mikkelsen, M. H. Solution-Processed Ultrafast, Room-Temperature Single-Photon Source at 1550 nm. *ACS Nano* **2025**, *19* (9), 10261–10272. https://doi.org/10.1021/acsnano.4c18261.

(s20) Zhao, S.; Lavie, J.; Rondin, L.; Orcin-Chaix, L.; Diederichs, C.; Roussignol, P.; Chassagneux, Y.; Voisin, C.; Müllen, K.; Narita, A.; Campidelli, S.; Lauret, J.-S. Single Photon Emission from Graphene Quantum Dots at Room Temperature. *Nat. Commun.* **2018**, *9*, 3470. https://doi.org/10.1038/s41467-018-05888-w.

(s21) Proppe, A. H.; Berkinsky, D. B.; Zhu, H.; Šverko, T.; Kaplan, A. E. K.; Horowitz, J. R.; Kim, T.; Chung, H.; Jun, S.; Bawendi, M. G. Highly Stable and Pure Single-Photon Emission with 250 ps Optical Coherence Times in InP Colloidal Quantum Dots. *Nat. Nanotechnol.* **2023**, *18* (9), 993–999. https://doi.org/10.1038/s41565-023-01432-0.

(s22) Yoshioka, M.; Yamauchi, M.; Tamai, N.; Masuo, S. Single-Photon Emission from Organic Dye Molecules Adsorbed on a Quantum Dot via Energy Transfer. *Nano Lett.* **2023**, *23* (24), 11548–11554. https://doi.org/10.1021/acs.nanolett.3c03279.

(s23) Rashi; Kaur, V.; Devi, A.; Bain, D.; Sen, T.; Patra, A. Probing the Fluorescence Intermittency of Bimetallic Nanoclusters Using Single-Molecule Fluorescence Spectroscopy. *J. Phys. Chem. Lett.* **2023**, *14* (45), 10166–10172. https://doi.org/10.1021/acs.jpclett.3c02823.
